# TSG-6 attenuates inflammation-induced brain injury via modulation of microglial polarization in SAH rats through the SOCS3/STAT3 pathway

**DOI:** 10.1186/s12974-018-1279-1

**Published:** 2018-08-20

**Authors:** Ran Li, Wenchao Liu, Jian Yin, Yunchang Chen, Shenquan Guo, Haiyan Fan, Xifeng Li, Xin Zhang, Xuying He, Chuanzhi Duan

**Affiliations:** Department of Neurosurgery, Zhujiang Hospital, The National Key Clinical Specialty, The Neurosurgery Institute of Guangdong Province, Guangdong Provincial Key Laboratory on Brain Function Repair and Regeneration, Southern Medical University, Guangzhou, 510282 China

**Keywords:** Subarachnoid hemorrhage, Early brain injury, Microglial polarization, TSG-6, Anti-inflammation

## Abstract

**Background:**

An acute and drastic inflammatory response characterized by the production of inflammatory mediators is followed by stroke, including SAH. Overactivation of microglia parallels an excessive inflammatory response and worsened brain damage. Previous studies indicate that TSG-6 has potent immunomodulatory and anti-inflammatory properties. This study aimed to evaluate the effects of TSG-6 in modulating immune reaction and microglial phenotype shift after experimental SAH.

**Methods:**

The SAH model was established by endovascular puncture method for Sprague–Dawley rats (weighing 280–320 g). Recombinant human protein and specific siRNAs for TSG-6 were exploited in vivo. Brain injury was assessed by neurologic scores, brain water content, and Fluoro-Jade C (FJC) staining. Microglia phenotypic status was evaluated and determined by Western immunoblotting, quantitative real-time polymerase chain reaction (qPCR) analyses, flow cytometry, and immunofluorescence labeling.

**Results:**

SAH induced significant inflammation, and M1-dominated microglia polarization increased expression of TSG-6 and neurological dysfunction in rats. rh-TSG-6 significantly ameliorated brain injury, decreased proinflammatory mediators, and skewed microglia towards a more anti-inflammatory property 24-h after SAH. While knockdown of TSG-6 further induced detrimental effects of microglia accompanied with more neurological deficits, the anti-inflammation effects of rh-TSG-6 were associated with microglia phenotypic shift by regulating the level of SOCS3/STAT3 axis.

**Conclusions:**

TSG-6 exerted neuroprotection against SAH-induced EBI in rats, mediated in part by skewing the balance of microglial response towards a protective phenotype, thereby preventing excessive tissue damage and improving functional outcomes. Our findings revealed the role of TSG-6 in modulating microglial response partially involved in the SOCS3/STAT3 pathway and TSG-6 may be a promising therapeutic target for the treatment of brain injury following SAH.

**Electronic supplementary material:**

The online version of this article (10.1186/s12974-018-1279-1) contains supplementary material, which is available to authorized users.

## Background

Subarachnoid hemorrhage (SAH) constitutes 5 to 10% of all strokes worldwide [1]. Healthy people can harbor intracranial aneurysms noted in occasional examination or emerging with several symptoms, otherwise detected with SAH onset [2, 3]. Once intracranial aneurysm ruptures, SAH is usually catastrophic because there exists no effective therapy applied in concomitant brain injury [4]. Currently, early brain injury (EBI) and delayed cerebral ischemia (DCI) represent the main effects of SAH at two stages, and neuroprotection and anti-vasospasm are the most studied targets in numerous research [5, 6]. Though many putative agents demonstrate exciting therapeutic effects, little has been translated into clinical application, and many have failed clinical trials [7]. Only nimodipine has been widely used in clinical application and has proven effective in the treatment of SAH [8].

SAH can induce several external stress events, including rapid ascension of intracranial pressure, sharp reduction in cerebral perfusion pressure, brain edema, and heme burden from red blood cell lysis, all of which converged to result in cellular pathophysiological changes [9]. Several studies have indicated that inflammation is invariably associated with brain damage after SAH [10–12]. As shown in clinical data, a sign of early inflammation in aSAH patients is correlated with poor neurological outcome on admission [13]. Microglia may sense even small imbalances of environmental homeostasis and are rapidly activated in a mode of dynamic morphology and polarization [14]. Afterwards, activated microglia appear to be the predominant source for a plethora of inflammatory mediators in the central nervous system (CNS) [15]. The progression of a dysfunctional and highly reactive microglial activation results in releasing high levels of pro-inflammatory and cytotoxic mediators that contribute to neuronal dysfunction and cell death [16]. The secreted cytokines together with environmental toxins and endogenous proteins, combined with neuronal death, further provoke unregulated activation of microglia resulting in the production of toxic factors that can propagate inflammation-induced neuronal death [17]. Therefore, alternative strategies based on a clearer comprehension of microglia-mediated inflammation are pressingly warranted. Accordingly, microglia, the prominent responder during neuroinflammtion, has been identified as the target in the present study.

Microglia polarization in different phenotypes exerting distinct responses is a central feature of disease progression involved inflammation [18]. Activated microglia are assumed to polarize into two extreme states: classical (M1, proinflammatory) and alternative (M2, anti-inflammatory) activation. Microglia dynamically transfer between M1 and M2 phenotypes after activation. Meanwhile, each phenotype can be identified by typical biomarkers. M1 phenotypic cells are characterized by the expression of CD86, CD68, iNOS, etc. Likewise, M2 phenotypic cells are associated with the expression of CD163, CD206, arginase-1(Arg-1), etc. In addition, M1 microglia are associated with a proinflammatory cellular state that has elevated expression of inflammatory cytokines, including IL-1β, TNF-α, and IL-6, which enhance brain damage. In contrast, M2 microglia release anti-inflammatory mediators, including IL-4, IL-10, and TGF-β, leading to beneficial neuroprotection [19, 20]. Though this M1/M2 paradigm is an oversimplified schema that simply divides the activated microglia populations into M1 and M2 phenotypes, it remains the most commonly used model to understand the role of microglia.

The phenomena of polarization of microglia has been carefully confirmed in several CNS diseases model. Fine-tuned microglia M1/M2 polarization has obtained positive therapeutic efficacy in experimental spinal cord injury (SCI), intracerebral hemorrhage (ICH), and ischemic brain injury models by suppressing the deleterious effects of inflammation, while boosting neuroprotective potential [21–23]. Largely, cues in the microenvironment and intertwined intracellular signals may determine how they polarize into M1 phenotype to exacerbate tissue injury or M2 phenotype to promote tissue recovery [24].

Recently, TSG-6 has emerged as a protective regulator against inflammation in cornea injury, peritonitis, autoimmune diabetes, asthma, and other inflammation-associated diseases. TSG-6 is a multifunctional glycoprotein composed of a hyaluronan-binding link domain and a CUB module in a contiguous fashion [25]. Normally, TSG-6 is upregulated in several pathological contexts especially related to inflammation. Collectively, the therapeutic effects of TSG-6 can be explained by participating in HA crosslinking and/or catalyzing the transfer of IαI heavy chains to HA [26]. It is, therefore, likely that TSG-6 acts as an endogenous inhibitor comprising part of a negative feedback loop in inflammation progression. Different from peripheral tissue, microglia play a major immune-response function instead of macrophages in the brain. However, the role of TSG-6 within CNS remains unclear. Our laboratory recently discovered that TSG-6 may preserve blood-brain barrier (BBB) by attenuating nitrative stress in an ICH model and improve outcomes in animal models of TBI by reducing the activation of microglia/macrophages [27, 28]. These results indicate that TSG-6 can play protective roles in CNS. However, whether TSG-6 impacts microglia polarization remains to be reported.

Herein, we aimed to address the possibility of TSG-6 in the regulation of microglia-mediated inflammation and the effects of TSG-6 on microglial polarization after SAH injury, as well as its potential mechanism in a rat endovascular puncture model of SAH.

## Methods

### Animals

Sprague–Dawley male rats (280-320 g) were obtained from the Animal Experiment Center of Southern Medical University. All experimental procedures and animal care were approved by the Southern Medical University Ethics Committee and were in accordance with the guidelines of the National Institute of Health. All rats resided in a light and temperature-controlled environment with ad libitum access to food and water and adapted to the environment 1 week before the experiments.

### Experimental design and animal groups

#### Time course and cell distribution

In this experiment, 84 male rats were divided into six groups at random (sham and, SAH 6, 12, 24, 48, 72 h after SAH). The mRNA and protein expression level and time course of TSG-6 were measured by qPCR and Western blot. Expression distribution was detected by FISH-ISH and double immunostaining to determine TSG-6 expression in different cell types of the brain in the sham group and 24 h after the SAH group. In additional study, qPCR and western blot were also employed to detect whether there was a statistical difference in TSG-6 gene and protein levels among the sham groups at 6 h, 12 h, 24 h, 48 h, and 72 h.

### Outcomes of treatment

To assess the role of TSG-6 on early brain injury after SAH, 102 rats were randomly divided into the sham group, SAH group, SAH + vehicle group, SAH + rh-TSG-6 (1 μg), SAH + rh-TSG-6 (5 μg), SAH + scrambled siRNA group, and SAH + TSG-6 siRNA group. All the rats were sacrificed 24 h after SAH according to the results of the first experiment. siRNA transfection efficiencies of each sample were verified using western blot analysis. Neurological scores, brain water content, and fluoro-Jade C (FJC) analysis were conducted.

### Correlation between TSG-6 and microglia polarization

To examine the effect of TSG-6 on microglia polarization, 60 rats were randomly assigned into the following groups: sham, SAH, SAH + vehicle, SAH + rh-TSG-6 (5 μg), SAH + scrambled siRNA group, and SAH + TSG-6 siRNA groups. Animals were sacrificed for brain tissue 24 h after SAH onsets. The samples were collected for qPCR, Flow cytometric analysis, ELISA, and immunofluorescence analysis. To clarify, we used samples partly from the first two for immunofluorescence (IF), qPCR, and WB experiments instead of having a separate cohort of SAH rats.

### Therapeutic mechanism of action

To explore the potential mechanism of TSG-6 on modulating microglia polarization, 36 rats were randomly assigned into the following groups: sham, SAH, SAH + vehicle, SAH + rh-TSG-6 (5 μg), SAH + scrambled siRNA, and SAH + TSG-6 siRNA groups. Immunofluorescence and western blotting was performed 24 h after SAH induction.

### Experimental SAH model

The SAH model was performed by endovascular puncturing for the induction of SAH as previously described [29]. Briefly, rats were deeply anesthetized by 1% pentobarbital sodium (40 mg/ kg, i.p.). A sharpened 4–0 nylon suture was inserted rostrally into the left internal carotid artery and perforated the bifurcation of the anterior and middle cerebral arteries until resistance was felt. Next, the suture was immediately withdrawn to allow blood reperfusion in the internal carotid artery, induced to SAH. Sham animals underwent the same procedures without vessel perforation.

### SAH grade

After euthanasia and removal of the brain, the basal brain was photographed immediately and divided into six segments as previously described [30]. Based on the amount of blood clotting, each area was blindly assigned a score from 0 to 3. All area scores were summed as the total SAH grade (maximum SAH grade = 18). Experimental rats with mild SAH whose SAH grades ≤7 were excluded from the study.

### Neurological score

The neurological scores were evaluated 24 h after SAH using the previously described modified Garcia scoring system [30]. Briefly, the evaluation included six tests scored from 0 to 3 or 1 to 3 and included the following: spontaneous activity, symmetry in the movement of four limbs, forelimbs outstretching, climbing ability, body proprioception, and the response to vibrissae stimulation. Possible scores ranged from 3 to 18. All the tests were evaluated by an observer who was blind to the treatment conditions. Higher scores represented better neurological function.

### Brain water content analysis

Brains were removed at 24 h after SAH and were divided into four parts: left hemisphere, right hemisphere, cerebellum, and brainstem. Left and right hemispheres were weighed immediately to obtain the wet weight and were then oven dried at 105 °C for 24 h to obtain the dry weight. The percentage of water content was calculated as follows: [(wet weight − dry weight)/wet weight] × 100%.

### Intracerebroventricular injection administration

In vivo transfection was performed as described previously [31]. TSG-6 siRNA (Santa Cruz Biotechnology, USA) and control scramble SiRNA (Santa Cruz Biotechnology, USA) transfection was performed with in vivo siRNA transfection reagent (Engreen Biosystem, Beijing, China) according to manufacturer protocols. TSG-6-siRNA was dissolved in RNase-free H_2_O at concentrations of 1 μg/1 μl; an equivalent concentration of scrambled-sequence siRNA was transfected into the negative control. Next, 5 μL TSG-6 siRNA or control siRNA was diluted with 5 μL in vivo transfection reagent. Finally, the mixture was injected intracerebroventricularly using a 10 μl Hamilton microsyringe (Microliter No. 701; Hamilton Company, Switzerland) under the guidance of a stereotaxic instrument (Stoelting Company, USA) under anesthesia. The SAH model was established 48 h later. rh-TSG-6 was dissolved in sterile PBS to a final concentration of 1 μg/10 μL or 5 μg/10 μL. Then, 1 μg or 5 μg rh-TSG-6 was infused into the cerebroventricle using a Hamilton syringe with the guidance of a stereotaxic instrument 1.5 h after SAH induction. The dosage of rh-TSG-6 was determined based on a previous study. The vehicle group were administered the same volume of sterile PBS or RNase-free H_2_O.

### Quantitative real-time polymerase chain reaction

Quantitative real-time polymerase chain reaction (qPCR) was performed and analyzed as previously described [27]. Total RNA from brain tissues with blood clots was extracted using TRIzol (Invitrogen, USA). Total RNA was reverse-transcribed to cDNA using the PrimeScript™ RT reagent Kit with gDNA Eraser (Takara, China). Rt-PCR reactions were performed on the Illumina-Eco Real-Time PCR Detection System (Gene Company Limited, USA) using the SYBR Premix Ex TaqII kit (Takara, China). The running procedure was 30 s at 95 °C, 40 cycles of 5 s at 95 °C, and 30 s at 60 °C, following a melt curve. Gene expression was quantified with standard samples and normalized with β-actin. Data were expressed as normalized messenger RNA (mRNA) expression (fold mRNA increase). The real-time PCR primer sequences are listed in Table 1.

**Table 1:** Real-time PCR primers used in this study

Primer name	Primer sequence	
TSG-6	Forward	CGTCTTGCAACCTACAAGCAGCTA
	Reverse	ACAGTTGGGCCCAGGTTTCA
CD86	Forward	GATTGCAGGTCCCAGTTCACTTC
	Reverse	CCACTGTCCTGCTTGGACTCAC
CD68	Forward	GGATTCAAACAGGACCGACAT
	Reverse	GGACACATTGTATTCCACTGCC
iNOS	Forward	TCCTCAGGCTTGGGTCTTGTTAG
	Reverse	TTCAGGTCACCTTGGTAGGATTTG
Arg-1	Forward	GCTGTGGTAGCAGAGACCCAGA
	Reverse	CATCCACCCAAATGACGCATAG
CD163	Forward	CTTTGGAATGGGCAAGAACAGAA
	Reverse	TGAGTGACAGCAGAGACGCTGA
CD206	Forward	TGGAGTGGCAGGTGGTTTATG
	Reverse	GGTTCAGGAGTTGTTGTGGGC
IL-1β	Forward	AATGCCTCGTGCTGTCTGA
	Reverse	GGATTTTGTCGTTGCTTGTCTC
IL-6	Forward	ATTGTATGAACAGCGATGATGCAC
	Reverse	CCAGGTAGAAACGGAACTCCAGA
TNF-α	Forward	TTCCAATGGGCTTTCGGAAC
	Reverse	AGACATCTTCAGCAGCCTTGTGAG
IL-4	Forward	TGCACCGAGATGTTTGTACCAGA
	Reverse	TTGCGAAGCACCCTGGAAG
IL-10	Forward	CAGACCCACATGCTCCGAGA
	Reverse	CAAGGCTTGGCAACCCAAGTA
TGF-β	Forward	CATTGCTGTCCCGTGCAGA
	Reverse	AGGTAACGCCAGGAATTGTTGCTA
β-actin	Forward	GGAGATTACTGCCCTGGCTCCTA
	Reverse	GACTCATCGTACTCCTGCTTGCTG

### Western blotting

The cerebral cortex tissues with blood clots were collected at corresponding time-points after SAH. Western blot (WB) was performed as described previously [27]. The following primary antibodies were used for WB: mouse anti-TSG-6 (Santa Cruz Biotechnology; 1:800), rabbit anti-STAT3 (Cell Signaling Technology; 1:2000), rabbit anti-phosphorylated STAT3 at Tyr705 (Cell Signaling Technology; 1:1000), rabbit anti-SOCS3 (Abcam; 1:1000), mouse anti-CD163 (AbD Serotec; 1:500), rabbit anti-CD86 (ProteinTech; 1:600), rabbit anti-IL-6 (PeproTech; 1:800), rabbit anti-IL-10 (ProteinTech; 1:600), and rabbit anti-β-actin (Cell Signaling Technology; 1:1000). The blot bands were quantitated by ImageJ software (National Institutes of Health, USA). Quantitative data were expressed as the target protein OD/β-actin OD ratio.

### Fluorescent in situ hybridization (FISH)

Paraffin-embedded brain slices were sectioned at 4 μm. Fluorescence in situ hybridization was performed using custom TSG-6-specific FISH Probes (Bersinbio, Inc., Guangzhou, China). Following manufacturer instructions, brain slices were hybridized with a TSG-6 mRNA FISH Probe and labeled with Alexa Fluor Cy3 (Life Technologies, Inc., USA). Immunohistochemistry was then performed using rabbit anti-Iba-1(Abcam; 1:500), rabbit anti-NEUN (Abcam; 1:400), and rabbit anti-GFAP (Abcam; 1:400). Alexa Fluor 488-conjugated IgG (1:200, Invitrogen; 1:200) was applied as a secondary antibody. Photos were taken with confocal microscopes (LSM800, Carl Zeiss, Germany) following manufacturer instructions.

### Immunofluorescence assay

Immunofluorescence staining was performed as previously described but with some modifications [27]. Briefly, brain sections were fixed in 4% paraformaldehyde for about 24 h. Coronal paraffin-embedded 4 μm thickness slices were conducted to Antigen retrieval and underwent blocking by 5% BSA for 1 h. After blocking, slices were incubated overnight at 4 °C with the following primary antibodies: mouse anti-NeuN (1:100, Millipore), rabbit anti-NeuN (1:400, Abcam), mouse anti-GFAP (1:300, R&D), rabbit anti-GFAP (1:400, Abcam), goat anti-Iba1 (1:300, Abcam), mouse polyclonal anti-TSG-6 (1:100, Santa Cruz Biotechnology), rabbit anti-CD163 (1:300, Abcam), rabbit anti-CD86(1:200, R&D), rabbit anti-pSTAT3 (1:400, Cell Signaling Technology), and rabbit anti-SOCS3 (1:500, Abcam). After washing with PBS, slices were then incubated with appropriate secondary antibodies for 1 h at room temperature. Following washing three times with PBS, the slices were re-stained by DAPI for 12 min before mounting. Then, images were obtained with a Leica DMi8 fluorescence microscope (Leica, Germany).

### Fluoro-Jade C (FJC) staining

FJC staining was used to investigate neurodegeneration. Sections were subjected to FJC staining in accordance with the manufacturer instructions. Briefly, the sections were immersed in a solution of 1% sodium hydroxide in 80% alcohol for 5 min, 70% alcohol for 2 min, distilled water for 2 min, and followed by 0.06% potassium permanganate for 10 min with gently shaking. The sections were immersed in a solution of 0.0002% FJC (Millipore Corporation, USA) in 0.1% acetic acid for 30 min. The sections were then rinsed three times in distilled water and allowed to dry at 50 °C for 15 min before covering with DPX medium (Sigma, USA). FJC-positive cell counting was performed as previously described but with modifications. Six sections located inside in the injured region were analyzed and FJC-positive cells were counted in each image. Data were presented by the average number of FJC-positive neurons in the fields as cells/mm^2^.

### Microglia isolation

Microglia of left cerebral cortex tissues with blood clots were isolated using a Percoll density gradient as described previously [32]. Briefly, brain samples obtained from each group after perfusion with 200 ml sterile saline were dissociated with 800 U DNase I (sigma, USA) and 7 ml Cell Dissociation Reagent (StemPro™ Accutase™, Gibco, USA) at 37 °C for 30 min in an incubator. After filtration with 70-μm cell strainers (BD Falcon, USA) to generate a single cell suspension, immune cells were separated by centrifugation using 40% Percoll in PBS at 1700 rpm for 30 min.

### Flow cytometry analysis

For flow cytometry analysis of microglial polarization status in the injured brain, the isolated microglia were stained with fluorescently labeled antibodies: CD11b-FITC (BD Biosciences), CD45-PE-Cy5 (BD Biosciences), CD163-APC (AbD Serotec), and CD86-PE (BD Biosciences) at 4 °C for 30 min. Flow cytometry was performed on a FACS VERSE apparatus (BD Bioscience) and obtained data were analyzed by Flow Jo software 7.6.1(Tree Star, USA).

### Inflammatory cytokine measurements

Total protein concentrations were measured using a BCA Protein Assay Kit (Genecopoeia, USA). Frozen brain samples were mechanically homogenized and centrifuged at 12,000 rpm for 15 min at 4 °C. The levels of interleukin-6 (IL-6), interleukin-10 (IL-10), tumor necrosis factor alpha (TNF-α), and interleukin-1β(IL-1β) were measured using specific ELISA kits (eBIOSCIENCE, USA) according to manufacturer instructions. The concentration of the cytokines was determined by color intensity measured by spectrometry in a micro ELISA reader (Varioskan Lux, Thermo Scientific). The results are expressed as picogram per milligram for tissue samples.

### Statistical analysis

All statistical analyses were performed using GraphPad Prism 6 (GraphPad software). Data are represented as a mean ± SD. Differences between two groups were analyzed with Student’s *t* test (two-tailed), and data were analyzed by one-way analysis of variance (ANOVA) with post hoc Tukey test or Dunnett’s test applied to assess multiple comparisons. Non-parametric data were analyzed using the Kruskal–Wallis H analysis followed by a Mann-Whitney *U* test. Statistical significance was set at a *P* value of < 0.05.

## Results

### No significant differences on TSG-6 mRNA expression and protein abundance were found in different time-points in sham

We found that there was no statistical difference of TSG-6 detected variables among sham groups at each time (Additional file 1A, B). Therefore, animals in sham group at 24 h after sham operation were chosen for further experiments.

### Both mRNA and protein level of TSG-6 were upregulated after SAH injury

**Fig. 1:**
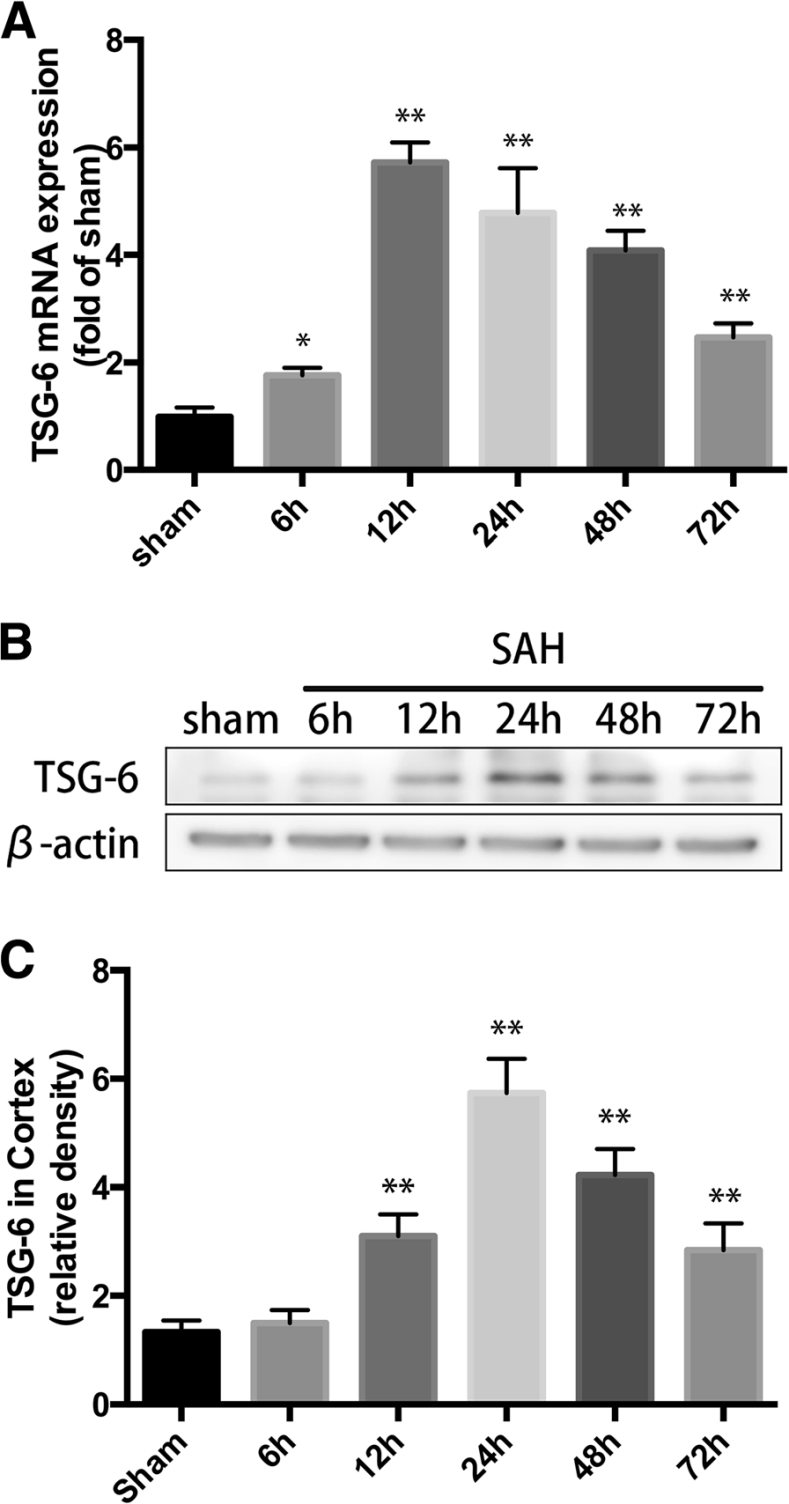
Endogenous expression of TSG-6 in brain tissue after subarachnoid hemorrhage (SAH). **a** Quantification of TSG-6 mRNA level in rat temporal cortex. **b** Western blot analysis showed the level of TSG-6 protein abundance at 6, 12, 24, 48, and 72 h after SAH. **c** Quantification of the TSG-6 protein level as shown in **b**. All values are presented as means ± SD, *n* = 6 in each time point per group. *p < 0.05, **p < 0.01 versus sham group

We first confirmed that brain TSG-6 increased in our SAH model. PCR and Western blot were performed to detect the time course of both mRNA and protein level of TSG-6 after SAH. The temporal profile of TSG-6 mRNA expression is shown in Fig. 1c. TSG-6 mRNA levels elevated immediately at 6 h after SAH (*p* = 0.0127) reached its peak value at 12 h, which almost six times that of the sham group. Afterwards, mRNA levels of TSG-6 gradually declined but were still significantly increased at 24 h, 48 h, and 72 h (*P* = 0.0022, *P* = 0.0001, *P* = 0.0016, respectively).

As shown in Fig. 1a, b, Western blotting revealed that the level of TSG-6 protein in the left temporal cortex increased over time. The level of TSG-6 protein abundance was weak in the sham group, while it increased significantly at 12 h (*P* = 0.0015), peaked at 24 h (*P* = 0.0008), and remained ascended at 72 h (*P* = 0.0011) post-SAH.

### The endogenous TSG-6 was mainly expressed in microglia after SAH injury

**Fig. 2:**
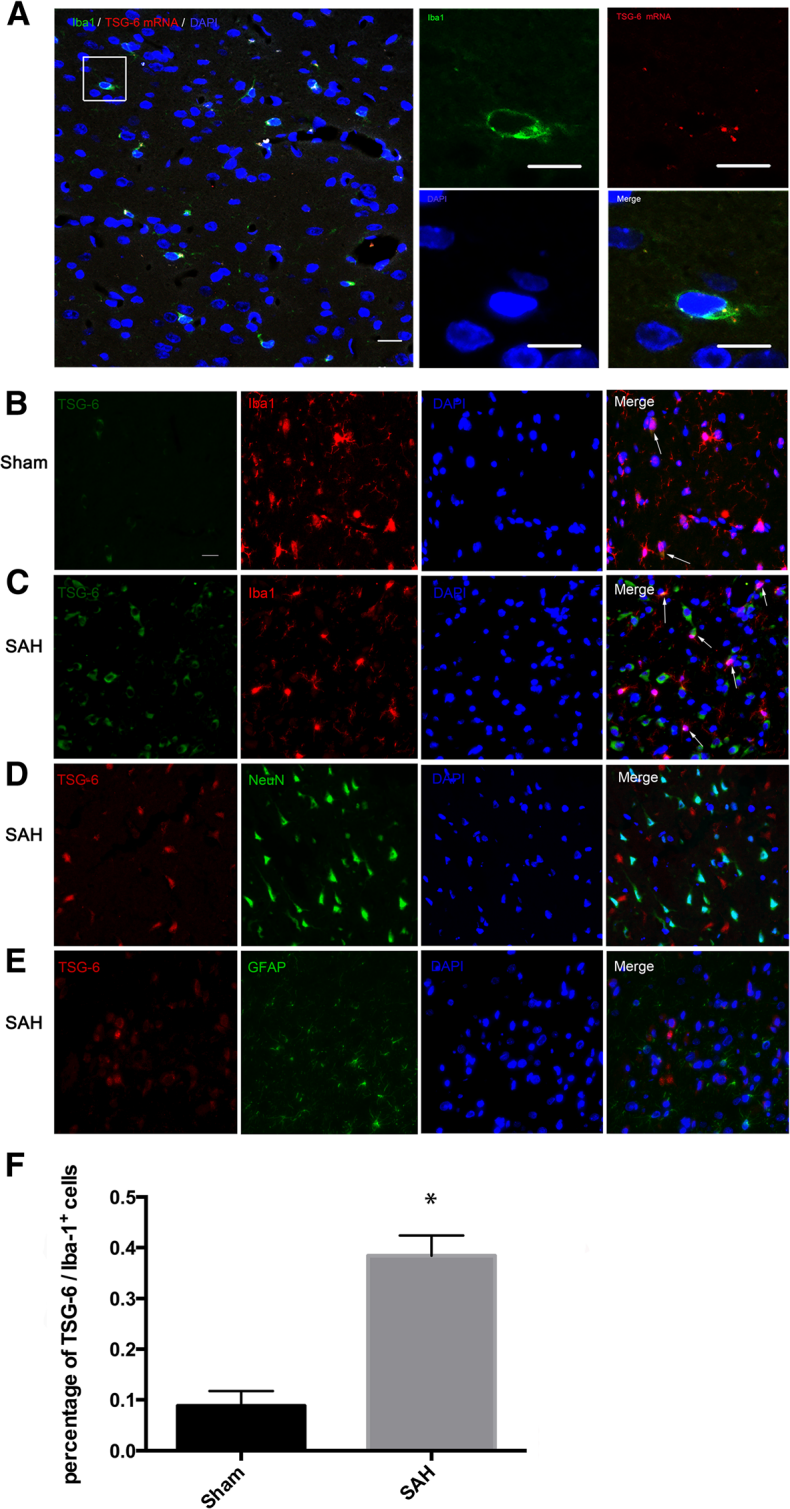
Spatial distributions of endogenous TSG-6 after SAH. **a** After injury, TSG-6 mRNA expression was clearly seen in microglia (Iba1^+^ cells). **b** Representative immunofluorescence staining slices of TSG-6 with calcium-binding adaptor molecule 1 (Iba1) in sham animals. **c** More Iba1-positive cells were observed after SAH when compared with sham group. Negative colocalization of TSG-6 with neurons (NeuN) (**d**) and astrocytes(GFAP) (**e**) at 24 h following SAH. Arrows point to TSG-6-positive microglia. *n* = 6 in each group. *p < 0.05 versus sham group. Scale bars = 20 μm. Bars in higher magnification panels are 10 μm

We used FISH-ISH and double-labeling immunofluorescence to identify the cell distribution of TSG-6 at the peak activation of TSG-6 (at 12 h according to the PCR and 24 h according to the WB). FISH-ISH analysis showed that TSG-6 mRNA was expressed in microglia (Fig. 2a) at 12 h after SAH. Double staining confirmed that a low TSG-6 protein abundance in the microglia was observed in the sham group. Compared with the sham group, SAH augmented the relative TSG-6 protein abundance predominantly in microglia (Fig. 2c), but not neurons (Fig. 2d) or astrocytes (Fig. 2e). The co-expression of TSG-6 and Iba-1 was obviously more after SAH (Fig. 2f).

### rh-TSG-6 alleviates SAH-induced brain injury (reduced brain water content and improved neurobehavioral outcomes) at 24 h after SAH

**Fig. 3:**
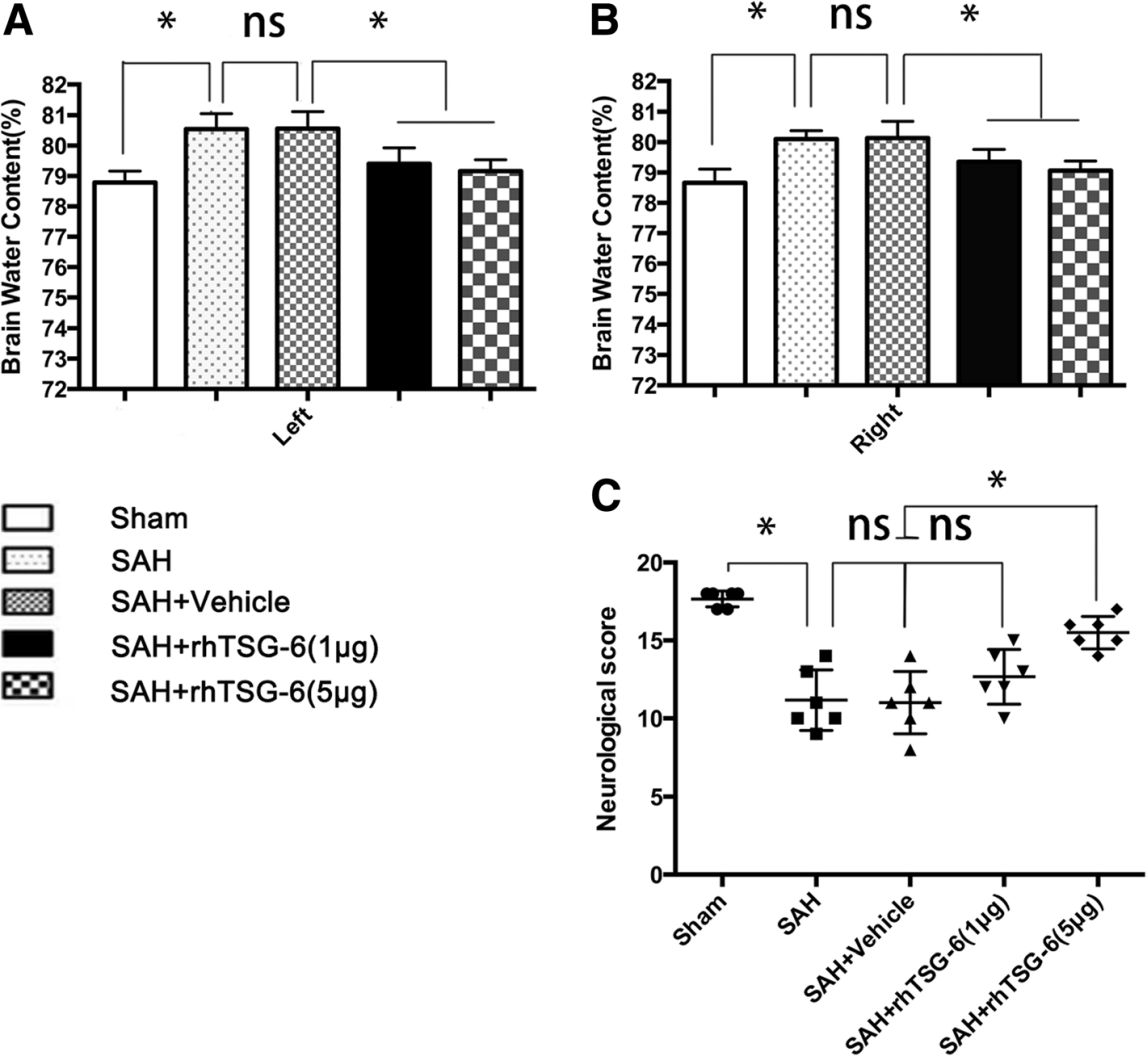
Effects of rh-TSG-6 treatment on brain edema (**a**, **b**) and neurobehavioral deficits (**c**) at 24 h after SAH. Brain water content using a wet/dry weight method was measured at 24 h after SAH. Neurological scores were recorded at 24 h after SAH. High dosage of rh-TSG-6 effectively reduced brain edema (**a**, **b**) and improved neurological functions (**c**) at 24 h following SAH. Values are expressed as the mean ± SD, *n* = 6 in each group. *P < 0.05

At 24 h after SAH, brain edema and neurobehavioral activity were examined in all groups. Two dosages of rh-TSG-6 (1 μg/10 μL and 5 μg/10 μL) were administrated intracerebroventricularly at 1.5 h after SAH. At 24 h, SAH insults induced poorer brain water content and neurological impairment compared to the sham group. No significant differences between SAH and SAH + vehicle groups in brain edema and neurological scores were observed. Both the two dosages of rh-TSG-6 dramatically ameliorated brain edema (Fig. 3a, b). However, only administration of high dosage rh-TSG-6 significantly improved neurobehavioral deficits (Fig. 3c) at 24 h post-ictus (*P* = 0.0022) vs. SAH + vehicle, *n* = 6. These results indicated that a high dosage is effective for reducing EBI; thus, a high dosage was selected for further studies.

### rh-TSG-6 treatment attenuated SAH-induced brain cell injury

**Fig. 4:**
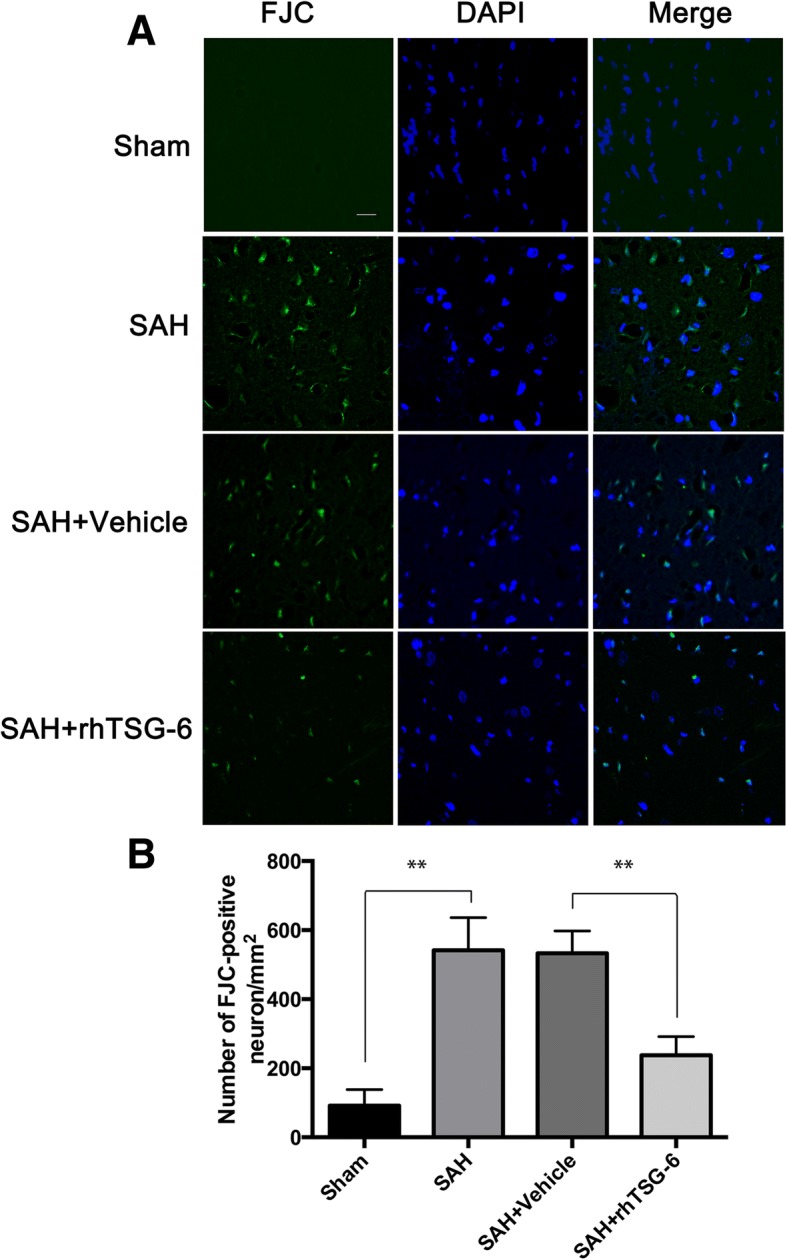
Effects of rh-TSG-6 on neuronal degenerating at 24 h after subarachnoid hemorrhage (SAH). **a** Representative microphotographs of Fluoro-Jade C staining (FJC)-positive neurons in the Sham, SAH, Vehicle and rh-TSG-6 groups at 24 h following operation. **b** Quantitative analysis of FJC-positive cells was performed at the ipsilateral cortex. *n* = 6 in each group. Data are expressed as mean ± SD. **P < 0.01. Scale bar = 20 μm

Neural loss is a key event in EBI after SAH. As shown in Fig. 4a, the procedure of SAH induced a large amount of neurodegeneration as revealed by FJC staining. Furthermore, there was no significant difference between the SAH and SAH + vehicle groups. When compared with the vehicle group at 24 h after SAH, the number of FJC^+^ cells was significantly decreased in the injured cortical region in rh-TSG-6-treated SAH rats (*P* = 0.0022) (Fig. 4a, b). Furthermore, the rh-TSG-6 group had a lower number of Fluoro-Jade C-stained neurons than the SAH-vehicle group at 72 h after SAH (*P* = 0.0002, Additional file 2A).

### Knockdown of endogenous TSG-6 aggravated neural death and neurologic deficits after SAH

**Fig. 5:**
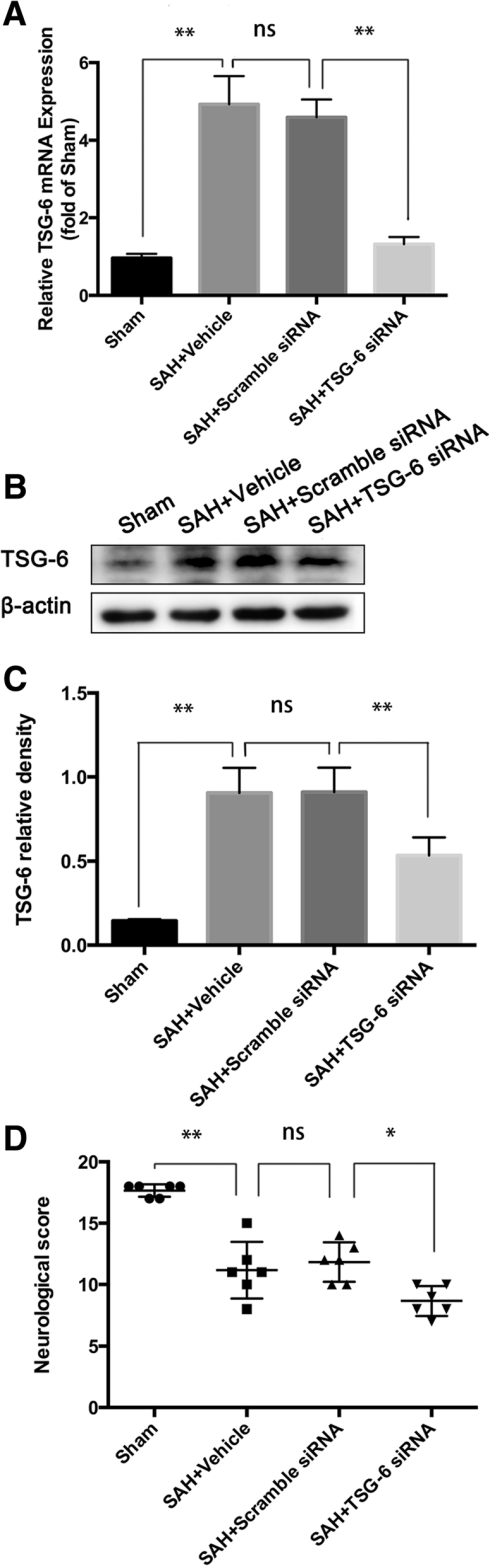
Effects of TSG-6 siRNA pre-treatment in SAH rats. The expression of TSG-6 at mRNA (**a**) and protein (**b**) level at 24 h after intracerebroventricular injection of TSG-6 siRNA. **c** Neurologic score evaluation was also performed at 24 h after SAH. Knockdown of endogenous TSG-6 significantly worsened neurologic deficits at 24 h after SAH. (*n* = 6 rats/group). Values are reported as means ± SD. *p < 0.05; **p < 0.01

To further explore the role of TSG-6 in the pathogenesis of EBI, knockdown of endogenous TSG-6 was performed. TSG-6 siRNA was injected into the cerebroventricle at 48 h before SAH induction. Interfering efficiency of TSG-6 siRNA was verified using qPCR and WB experiments. Neurologic score evaluation was also performed 24 h after SAH. As shown in Fig. 5a–c, scramble siRNA had no effect on endogenous TSG-6 mRNA and protein expression compared to the SAH group, but knockdown of endogenous TSG-6 with siRNA decreased the mRNA and protein level of TSG-6 in the brain (*p* = 0.0001 and *p* = 0.0004, respectively; *n* = 6; *n* = 6). Furthermore, knockdown of endogenous TSG-6 significantly aggravated neurologic deficits 24 h after SAH (Fig. 5d).

### SAH-induced activation of microglia and microglial M1 phenotype polarization was dominated during the SAH early phase

**Fig. 6:**
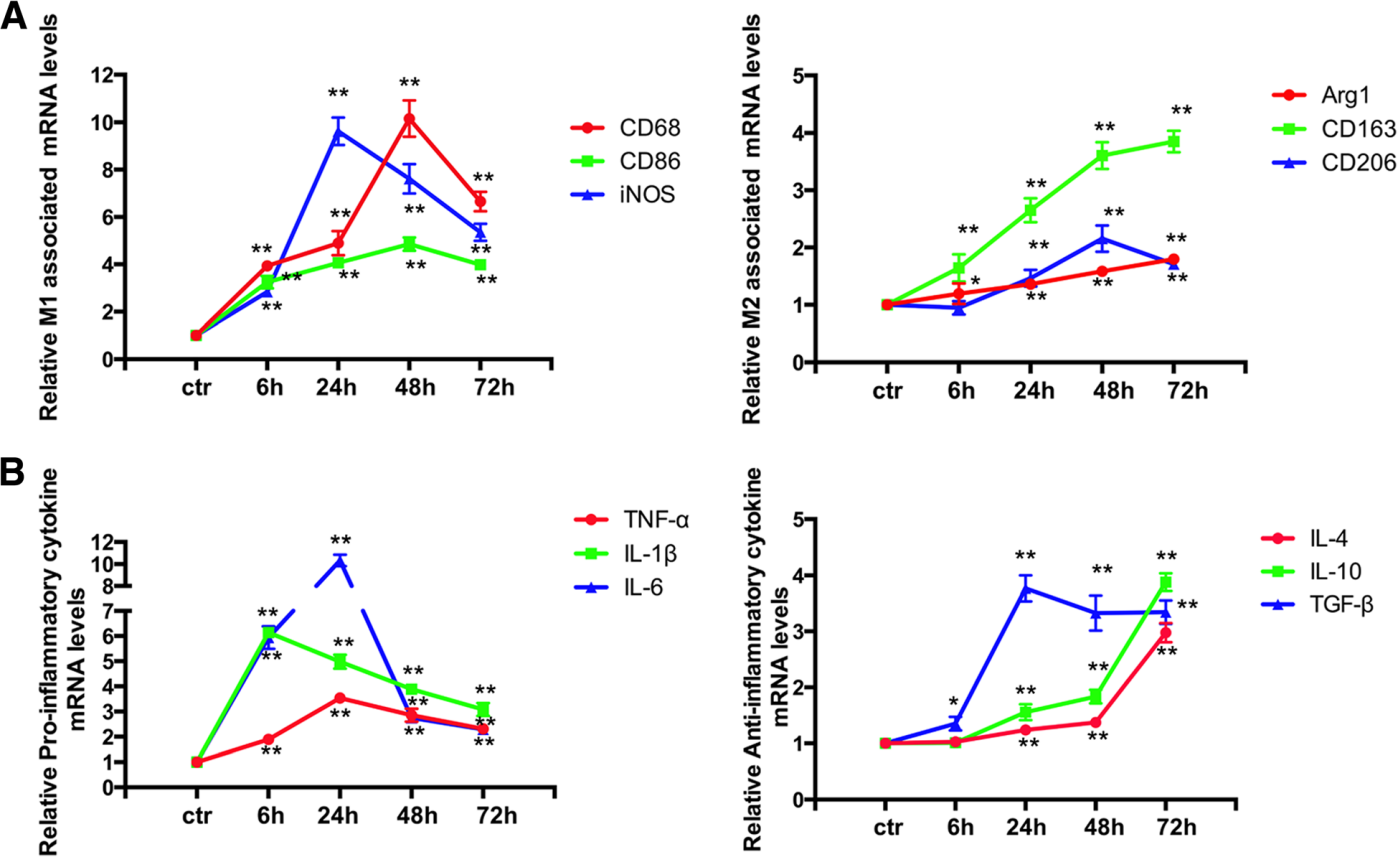
Microglial phenotypic characteristics after SAH induction. **a** M1/M2 Markers of activated microglia increased over time after SAH. **b** Time course changes of mRNA expressions at 6 h, 24 h, 48 h, and 72 h for proinflammatory cytokines (TNF-α, IL-1β, IL-6) and anti-inflammatory cytokines (IL-4, IL-10, TGF-β) after SAH. Samples for PCR experiment sorted from left temporal brain lysates. Quantifications are expressed as the fold change compared to the sham group. *n* = 5 in each group. *P < 0.05 and **P < 0.01 vs. sham group. Ctr represents sham group

Activation of microglia is a hallmark of neuroinflammation. In this study, we investigated the characteristics of microglial activation in the early stages following SAH induction. We first detect whether microglia were markedly activated after SAH. Microscopic examination of whole brain sections stained for Iba1 confirmed that SAH-induced microglia were dramatically activated for whole brain sections compared to the sham group (Additional file 3). Next, we detected mRNA levels of markers of M1/M2 microglial cells and M1/M2-associated cytokines at corresponding times post-SAH. The data showed M1-associated markers CD68, CD86, and iNOS increased immediately and peaked at 24 h (iNOS) and 48 h (CD68, CD86) and remained at high levels for 72 h. M2-associated markers increased slowly and peaked at 48 h (CD206) and 72 h (Arg-1, CD206) and maintained a slight growth within 72 h after SAH (Fig. 6a).

Consistent with the M1/M2 marker qPCR results, mRNA expression levels of both pro-inflammatory and anti-inflammatory cytokines showed similar trends. Generally, the pro-inflammatory cytokine expression levels had a striking burst and peaked at 6 h (IL-1β) or 24 h (TNF-α, IL-6) post-SAH. In contrast, expression of anti-inflammatory cytokines was not augmented significantly until 6 h (TGF-β) or 24 h (IL-4, IL-10) and maintained a relatively gentle increase (Fig. 6b).

These results demonstrated that SAH induced a remarkable increase in both M1- and M2-type mRNA expression in the injured brains of rats compared with the sham groups. Moreover, SAH-induced activated microglia were mainly polarized to the M1 phenotype.

### TSG-6 treatment provides neuroprotection in the brain under SAH conditions by modulating microglial phenotype and decreasing its pro-inflammation activity

**Fig. 7:**
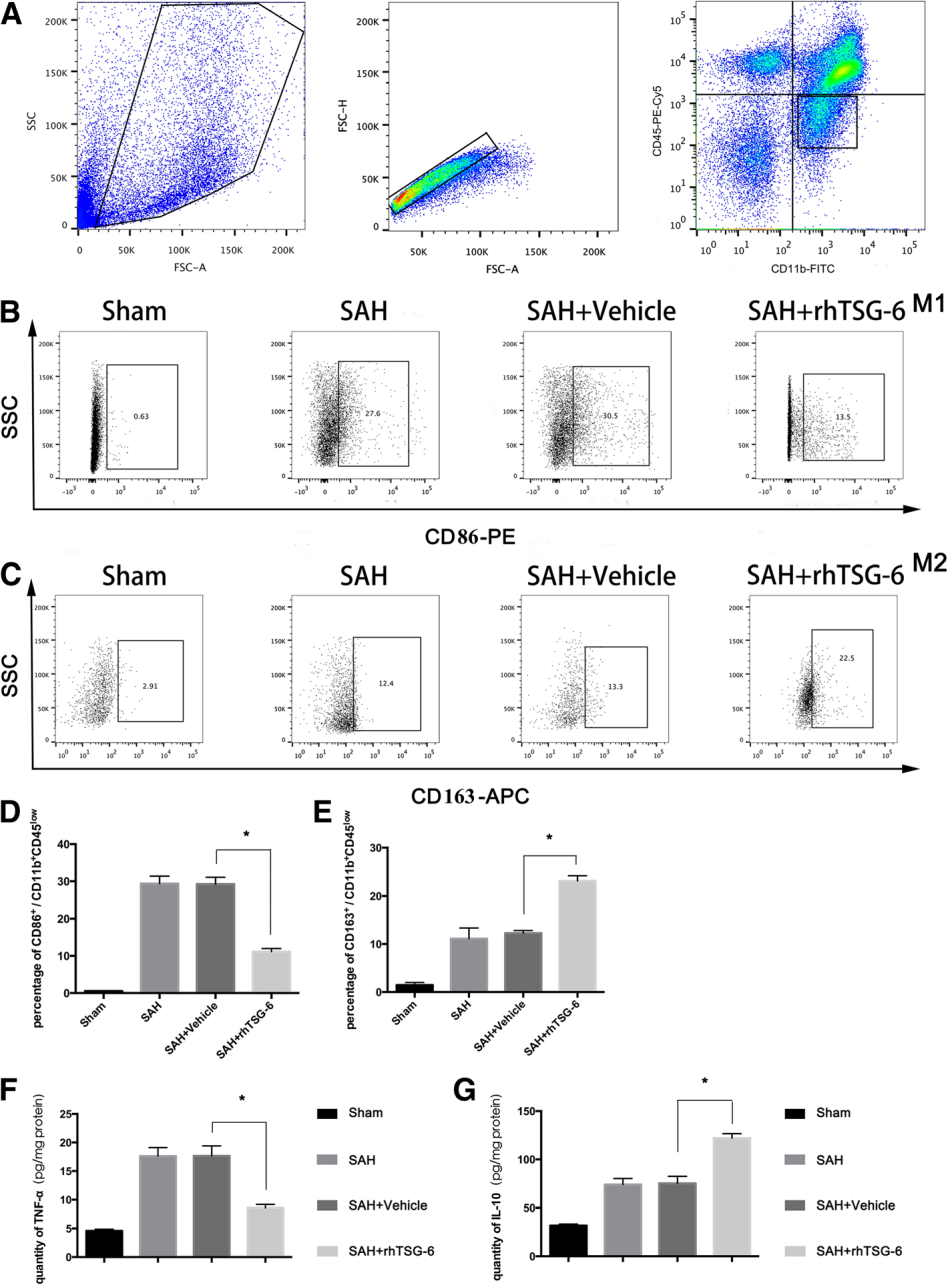
Effects of rh-TSG-6 on SAH-induced microglia polarization. **a** Representative FACS plots showing gating strategy we use in flow cytometry analysis. Cells populations in the right dot plots defined as CD11b^+^ CD45^low^ (microglia) were gated for further analysis. Representative dot plots showing the ratio of CD86-positive subsets (**b**) and CD163-positive subsets (**c**) in CD11b^+^ CD45^low^ populations in the sham group, SAH group, SAH + vehicle group and SAH + rh-TSG-6 group. Bar graphs show quantitative analysis of CD86-positive subsets of CD11b^+^ CD45^low^ populations (M1 microglia) (**d**) and CD163-positive subsets of CD11b^+^ CD45^low^ populations (M2 microglia) (**e**) in different groups. Inflammatory mediators expressed in the brain cortex after SAH were detected with ELISA. Tissue TNF-α and IL-10 concentrations were analyzed from samples and standards in duplicates and expressed as pictograms per milliliter. rh-TSG-6 administration obviously reduced the level of TNF-α (**f**) and increased the level of IL-10 (**g**) in the brain tissue. Samples for FACS and ELISA experiments sorted from tissue lysates of left temporal lobe of the brain. The values (means ± SD) are representative of three independent experiments. *p < 0.05. *n* = 6/group. SSC = side scatter channel, FSC-A = forward scatter channel area, FITC = fluorescein isothiocyanate, PE = phycoerythrin, and APC = allophycocyanin

Polarization of microglia was involved in the pathological progression of neurotoxic insults and strokes. To assess whether TSG-6 has a biological role in microglial polarization, the ipsilateral temporal lobe cortex was subjected to flow cytometry for the ratio of M1- and M2-type microglia measurement. Using flow cytometric analysis [33], we distinguished between activated microglia (CD11b^+^ CD45^low^) and infiltrating macrophages (CD11b^+^ CD45^high^) and detected changes in classical activation marker CD86 and alternative activation marker CD163 expression in specific cell populations. The gating strategy is shown in Fig. 7a. As shown in Fig. 7b, compared with the sham groups, the SAH and vehicle-treated group showed a higher percentage of CD11b^+^ CD45^low^ microglia labeled with markers for M1 (CD86) and M2 (CD163) phenotypes. In rats treated with rh-TSG-6 after SAH induction, the expression of CD163 was increased on CD11b^+^ CD45^low^ microglia than those in the vehicle treated group, while the expression of M1 marker (CD86) exhibited a remarkable attenuation. In accordance with declined M1 polarization elevating M2 phenotype after rh-TSG-6 treatment, there was a remarkable attenuation of TNF-α expression level and upregulated IL-10 expression level measured by ELISA in the rh-TSG-6 treatment group. As expected, no significant changes of either M1 or M2 ratio were noticed between the SAH and vehicle-treated groups.

Overall, these results demonstrated that rh-TSG-6 treatment partially retained SAH-driven M1 polarization and skewed the balance of M1/M2 ratio to a beneficial phenotype, thereby conferring its neuroprotective properties.

### Deficiency of endogenous TSG-6 leads to an exaggerated pro-inflammatory microglial phenotype

**Fig. 8:**
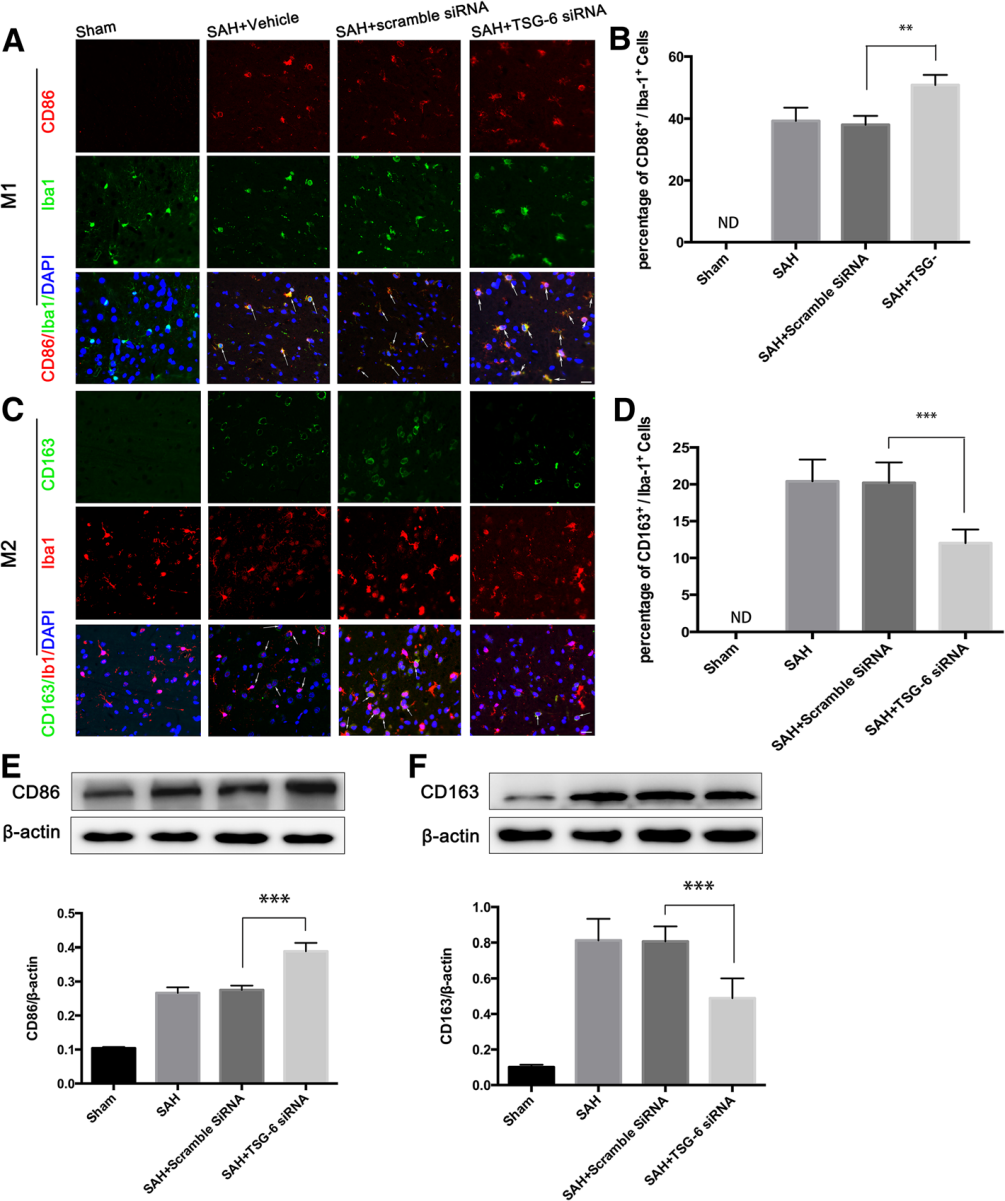
Deficiency of endogenous TSG-6 leads to an exaggerated proinflammatory microglial phenotype. Immunofluorescence labeling and quantification analysis (**a**–**d**) and western blot (**e**, **f**) and showing that inhibition of TSG-6 further enhanced the increased levels of CD86 and the enhanced CD163 levels was decreased 24 h after SAH compared with the scramble siRNA group. The samples for western blot are tissue lysates obtained from the left temporal lobe of the brain. White arrows indicate typical cells. Values of the relative densitometric analysis are expressed as mean ± SD, Scale bar = 20 μm, *n* = 6 in each group. **P < 0.01; ***P < 0.001

To further determine the role of TSG-6 in modulatory effects of triggering phenotypic conversion of microglia, mice were subjected to TSG-6 siRNA treatment 48 h before SAH induction. The results of double immunofluorescent staining and western blot showed that the M1-associated marker CD86 (Fig. 8a, b, e) and M2-associated marker CD163 (Fig. 8c, d, f) were both significantly upregulated 24 h after SAH compared to the sham group. When TSG-6 was suppressed, the induction of SAH potentiated the protein expression of CD86, while the expression of M2 marker CD163 in Iba1 was lower than the scramble siRNA-treated group.

Considering the above results, we concluded that a loss of TSG-6 triggers microglial phenotypic changes with a bias towards pro-inflammatory activity, namely amplifying M1 polarization of activated microglia.

### Effects of TSG-6 were involved in impeding the procedure of phosphorylation and the cellular location of Stat3

**Fig. 9:**
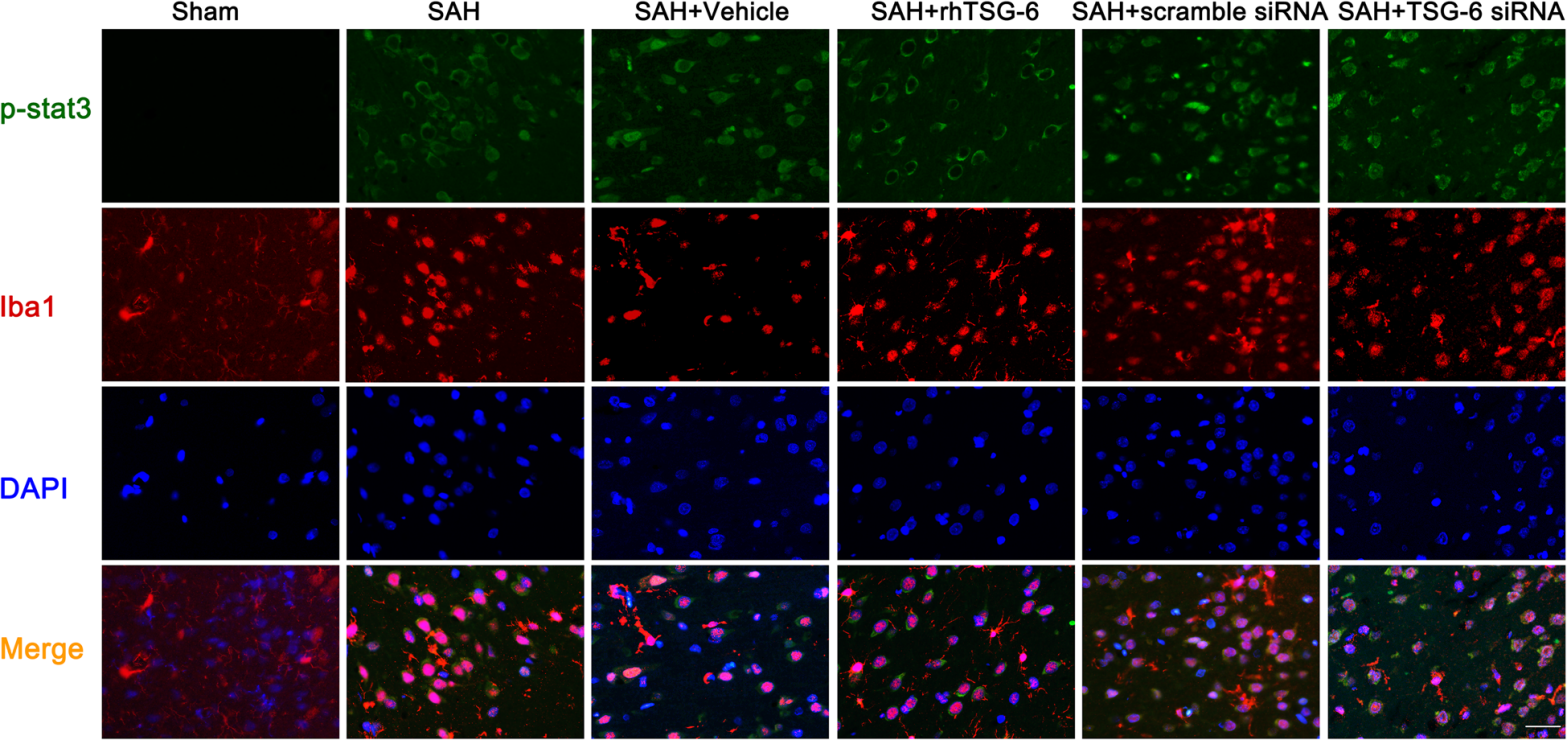
The effects of rh-TSG-6 on phosphorylation of Stat3 and cellular location of p-STAT3. The phosphorylation of STAT3 was assessed by immunofluorescent double-labeling. Immunoreactivity for p-STAT3 is attenuated at 24 h in the SAH + rh-TSG-6 group, as compared with the SAH + vehicle group. TSG-6 deficiency increased the expression of p-STAT3 and more translocation from the cytosol to the nucleus occurs was observed. Scale bar = 20 μm

STAT3 is an important transcription factor associated with microglial polarization status. Overactivation of p-STAT3 can contribute to the upregulation of proinflammatory cytokines under some kinds of stroke. Since the activity of pSTAT3 was observed mainly in microglia/macrophages, but not in either neurons or astrocytes, therefore, immunofluorescence determined the expression of p-STAT3 in microglia in the rat cortex following SAH at 24 h. As shown in Fig. 9, fluorescence intensity of p-STAT3 was barely detected in the sham group. Obviously, rats which underwent SAH exhibited significantly increased p-STAT3 levels compared with sham-operated rats. Next, intervention with rh-TSG-6 diminished the level of phosphorylation of STAT3 in the SAH group. Correspondently, phosphorylation level of p-STAT3 was more enhanced in TSG-6 siRNA-pretreated rats. In addition, a reduction in nuclear translocation of STAT3 following rh-TSG-6 treatment and p-STAT3 immunoreactivity were more elevated following silencing treatment. Therefore, these results suggested that TSG-6 may be associated with the phosphorylation of STAT3.

### The effects of TSG-6 on modulating microglial phenotype was involved in SOCS3/STAT3 pathway

**Fig. 10:**
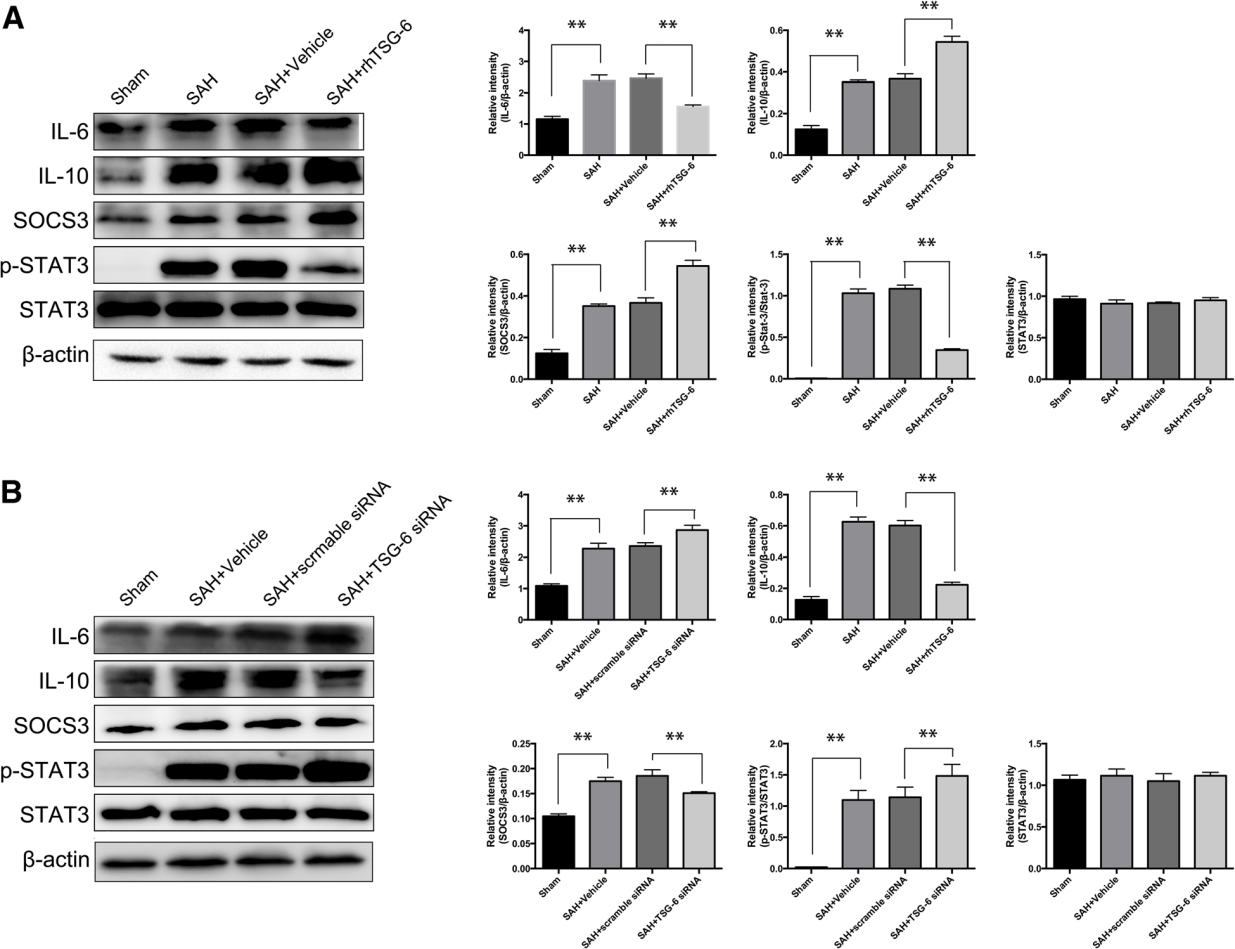
Effects of rh-TSG-6 and TSG-6 siRNA treatments on SOCS3/STAT3 axis. **a** Effect of exogenous TSG-6 on STAT3 pathway expression was evaluated by western blot and the relative IL-6, IL-10, SOCS3, p-STAT3, and STAT3 densities were quantificationally evaluated. **b** The representative western blot analysis of STAT3 axis in SAH rats accepted intracerebroventricular injection of siRNA as indicated and quantification of the level of IL-6, IL-10, SOCS3, p-STAT3 and STAT3 after deficiency of TSG-6. Data are expressed as the mean ± SD from six rats. **P < 0.01

Among the candidate signal pathways, the STAT3 signaling pathway can play a pivotal role in neuroinflammation and the activation of microglia within CNS. To further explore the underlying mechanism of TSG-6 in regulating microglia activation, factors in the STAT3 pathway pivotal to microglia polarization were tested. Results of Western blot (Fig. 10a) showed that after SAH, inflammatory responses occurred. Significant activation of STAT3 was observed accompanied with elevated expression of IL-6 rather than IL-10 and a raised SOCS3 levels in comparison with the sham group. However, the induction of SAH or TSG-6 was not observed to change the protein level of STAT3. Additionally, post-SAH TSG-6 upregulation was associated with an increase in IL-10 and SOCS3 levels and a decrease in IL-6 and phospho-STAT3 levels in brain tissues 24 h after SAH. IL-6-mediated activation of STAT3 was inhibited in injured brain tissues of SAH rats treated with rh-TSG-6.

Finally, we knocked down endogenous TSG-6 to test the association between TSG-6 and the SOCS3/STAT3 axis (Fig. 10b). Conversely, deficiency of TSG-6 in SAH rats showed more increased pSTAT3 compared to scramble siRNA treated rats. As expected, the two inflammatory cytokines indicated opposite trends (amplifying IL-6 expression and declining IL-10 expression) contributed by TSG-6 silencing treatment. Expression of SOCS3 showed a weaker response to SAH insult. Therefore, these results verified TSG-6 functions in altering microglial phenotypes by modulating SOCS3/STAT3 axis expression in inducible SAH rat model.

## Discussion

In this study, we revealed a neuroprotective effect with emphasis on targeting the immunomodulatory functions afforded by TSG-6 in a rat model of SAH. Our data demonstrated that TSG-6 was upregulated in the brain cortex in a time-dependent manner after SAH. To further determine the role of TSG-6 following SAH, we continued our research in experimental rats. Exogenous TSG-6 greatly attenuated neurological deficits and brain water under inducible subarachnoid hemorrhage in vivo. After silencing endogenous TSG-6, early brain injury was further exacerbated. These results indicated that the neuroprotective effect of TSG-6 is associated with phenotypic modulation of the microglia shift. Signaling protein expression-associated STAT3 in brain tissue after SAH were investigated. The finding showed that levels of TSG-6 were involved in STAT3 phosphorylation, which was an important factor for controlling microglial inflammatory subtypes. Accordingly, these results suggested that TSG-6 may play an endogenous brain protection role after SAH and an enhanced expression or supplementation of TSG-6 may induce a selective modulation of microglia polarization to anti-inflammatory phenotypes via the SOCS3/STAT3 pathways (Fig. 10).

TNF-stimulated gene-6 (TSG6) is a pleiotropic immunomodulatory protein activated rapidly in response to stimuli and functions at an early phase of inflammatory procedure. The protective effects of TSG-6 have been well studied in the brain, most of which focused on the anti-inflammatory aspect, especially its role in inhibiting activation of microglia/macrophages [27, 28, 34]. Given that the suppression of microglia/macrophages may also compromise the normal physiological defense mechanism of CNS and induce inevitable side effects [35, 36], the mechanism by which TSG-6 confers neuroprotection requires further investigation. In this study, the protein of TSG-6 was almost located in Iba1^+^ microglia. However, this result was different from Coulson-Thomas et al.’s work, in which they found TSG-6 was only located in astrocytes in a SCI rat model [37]. As indicated in Fig. 2c, TSG-6 staining is positively co-expressed in not only microglia but also other cell types as well. According to the literature, blood-derived immunocytes, such as neutrophils and DC cells, can express TSG-6 under inflammation stimulation [26]. Therefore, under the circumstance that BBB is no longer intact after SAH insult, TSG-6 could originate from infiltrating peripheral immunocyte. Indeed, we cannot exclude the possibility that TSG-6 expressed by other cells also plays a role, but the proportion of infiltrating peripheral immune cells is relatively small compared to resident microglia, especially in the early stages [38, 39]. Thus, we assumed that data detected in the current study mainly reflects the biological function of microglial TSG-6. Correspondingly, by intervening in the concentration of TSG-6 (exogenous supplementary or knockdown), our results indicated that the level of microglial TSG-6 was closely associated with the severity of brain injury after SAH, which was in lines with several other studies that the level of TSG-6 can reflect the disease progression and act as a novel prognostic factor [40].

Some may argue that simply dividing microglia into the supposed dichotomy between M1 and M2 phenotypes is oversimplified [41]; however, this classification may provide a bridge for understanding the function of microglia in several brain diseases [24]. When irritated proinflammatory-status microglia dominates the inflammatory microenvironment, inflammation-induced developmental brain injury can be observed in some CNS diseases [42, 43]. Consistent with the findings of previous study [44], our study showed that, after induction of SAH, augmented microglia were identified by the panorama of brain slices and more strikingly elevated proinflammatory genes compared to anti-inflammatory genes within 72 h after SAH was revealed by PCR. rh-TSG-6 treatment significantly suppressed the prevailing M1 phenotypes and observed a trend towards M2 phenotypes (Fig. 4). Resident microglia are major cells involved in inflammatory processes in the brain and are first to respond to the disturbed brain environment [38, 45]. Yet the time for microglia-mediated inflammation response to manifest occurred over hours to days, thereby creating an opportunity for clinical therapeutic intervention [46]. Accordingly, early limitation of microglia skewing towards proinflammatory phenotypes can help to preserve brain tissue. As expected, a lower abundance of endogenous TSG-6 protein led to the provocation of M1 phenotypic microglia after SAH and to be concomitant with the marked proinflammatory environment are degenerated neurons and upcoming unfavorable outcomes, while after administration of rh-TSG-6, the modified inflammatory milieu at the early stage of EBI through M1/M2 switching was accompanied by an improved outcome. A timely shift to beneficial M2 microglia can resolve neuroinflammation and create a microenvironment friendly to CNS repair [21]. However, long-term blockage of inflammation via suppressing M1 activation may not induce overall beneficial effects because deficiency of M1 phenotypic microglia will hinder clearing cell debris so as to prolong the inflammatory process. It is clear that cessation of inflammation progression before mounting to its peak is effective in alleviating brain injury. Accordingly, in the present study, we observed the effect of acute rh-TSG-6 treatment on early outcomes (24 h) in SAH rats. Modulation of the M1/M2 balance by TSG-6 is protective against neuronal injury 24 h after SAH. This effect also has a beneficial impact on neuronal survival at 72 h after SAH as indicated by our FJC data. Collectively, these results indicated a causal relationship between TSG-6 and the M1/M2 microglia switch.

Controlled neuroinflammation and immune cell infiltration within injured CNS were supposed to serve protective and beneficial functions [47]. However, to what extent and to what time inflammatory processes are deleterious and/or beneficial to brain recovery remain controversial. Therefore, a more precision modulation of microglial inflammation response remains to be explored. To date, there exists much uncertainty over which intracellular signaling pathways are involved in the mediation of the microglial shift in SAH. It is reported that STAT family members play various roles in microglial and macrophage polarization [48]. Our study discovered that microglial STAT3 was highly activated in the hemorrhagic hemisphere after experimental SAH. However, disparate functional outcomes from activated STAT3 were observed in different cerebral pathological conditions. Furthermore, whether microglial STAT3 activation during a pathologic condition results in inflammation or neuroprotection remains relatively controversial [49, 50]. Recent studies have shown that upstream kinase can regulate STAT3 and the subsequent downstream genes regulated by STAT3 [51]. Specifically, it has been demonstrated that both the proinflammatory mediator IL-6 and the anti- inflammatory cytokine IL-10 share the same STAT3 pathway [52]. Depending on different stimuli, IL-10-mediated STAT3 responses can lead to an anti-inflammatory phenotype associated with a higher expression of M2 neuroprotective markers [53]. However, under some sublethal strikes, a loss of normal feedback modulation on STAT3 from SOCS3 can result in overactivation of STAT3, thereby favoring IL-6 driven STAT3 [54, 55]. In good agreement with the previous studies [56], our results demonstrated that overactivation of STAT3 in microglia is detrimental, and a reduced phosphorylation of STAT3 may provide anti-inflammatory effects.

Regarding the possible mechanisms underlined in TSG-6-conferred neuroprotection, we discovered that treatment with rh-TSG-6 following SAH effectively decreased the expression of p-STAT3 accompanied by the increased of expression of SOCS3 as well as IL-10 and a decreased IL-6 expression related to the vehicle-treatment group, both of which correlated with a dominated anti-inflammatory microenvironment. In vivo TSG-6 knockdown further aggravated STAT3 activation and reversed the effects of IL-6, IL-10, and SOCS3 expression. As a result, a pronounced polarization to M1 phenotype microglia was linked with worsened brain injury, which is consistent with several previous works [23, 57]. Taken together, we speculated that TSG-6 appeared to play a unique role in M1/M2 polarization by modulating IL-6/STAT3 or/and IL-10/STAT3 activity through SOCS3 activation.

Recently, a study from Mittal et al. supported our finding whereby TSG-6 inhibited aberrant activation of STAT3 and primed the innate immunity response from a state of generating proinflammatory cytokines to a distinct anti-inflammatory status [58]. Although we have demonstrated that TSG-6 confers its neuroprotection partly depending on the SOCS3/STAT3 axis, the detailed mechanisms underlined require further investigation. Additionally, this study mainly focused on the role of TSG-6 in the early stage of SAH injury. The potential long-term roles of TSG-6 on M1/M2 phenotypic balance and responses to brain injury are not addressed herein and should be investigated in the future studies. Admittedly, injury-recovery is a continuously variable process. All immunotherapies may not achieve optimal clinical transformation if they do not compliment changes during the overall process. Likewise, whether the effect of TSG-6 beyond the EBI at a later time satisfies the demands of the CNS microenvironment will impact the recovery processes and the final neurological state. However, due to limitations inherit in the puncture SAH model, it is difficult to explore this research topic. A more perfect SAH model which is suitable for long-term research on SAH pathophysiological conditions is required.

## Conclusions

In summary, we identified the protective effects of TSG-6, a novel endogenous anti-inflammation protein, after SAH insults by inhibiting inflammation through the SOCS3/STAT3 pathway. It was discovered that exogenous administration of rh-TSG-6 can provide neuroprotective effects related to modulating microglial polarization phenotypes. Our finding suggested that TSG-6 is a promising candidate for alleviating the degree of EBI following SAH, which may expand application in targeting microglial overactivation-mediated inflammation in cerebrovascular diseases.

## Additional files

Additional file 1: Analysis of levels of TSG-6 gene and protein in different time-points in the sham (6 h, 12 h, 24 h, 48 h, 72 h). No significant differences of TSG-6 gene (A) and protein (B) were found among different groups. All values are presented as means ± SD, *n* = 4 in each time point per group. (TIF 532 kb)

Additional file 2: Effects of rh-TSG-6 on neuronal degenerating at 72 h after subarachnoid hemorrhage (SAH). Representative microphotographs and quantitative analysis of Fluoro-Jade C staining (FJC)-positive neurons in the sham, SAH, vehicle and rh-TSG-6 groups at 72 h following operation. *n* = 6 in each group. Data are expressed as mean ± SD. ***P < 0.001. Scale bar = 20 μm. (TIF 856 kb)

Additional file 3: Microglial phenotypic characteristics after SAH induction. Photomicrograph of microglia(red) and DAPI(blue) double immunostaining in a coronal section of the whole brain in sham and SAH groups. *n* = 5 in each group. Scale bar = 2000 μm. (TIF 1507 kb)

## Abbreviations

APC: Allophycocyanin
CNS: Central nervous system
EBI: Early brain injury
FITC: Fluorescein isothiocyanate
FJC: Fluoro-Jade C
GFAP: Glial fibrillary acidic protein
Iba1: Ionized calcium-binding adapter molecule 1
ICH: Intracerebral hemorrhage
NeuN: Neuronal nuclear protein
PE: Phycoerythrin
qRT-PCR: Quantitative real-time polymerase chain reaction
SAH: Subarachnoid hemorrhage
SCI: Spinal cord injury
SD: Sprague–Dawley
siRNA: Small interfering RNA
SOCS3: Suppressor of cytokine signaling 3
STAT3: Signal transducer and activator of transcription 3
TSG-6: TNF-α stimulated gene/protein 6
