# Supplementary figures and images for: TSG-6 attenuates inflammation-induced brain injury via modulation of microglial polarization in SAH rats through the SOCS3/STAT3 pathway
Source: J Neuroinflammation. 2018 Aug 20;15:231. doi: 10.1186/s12974-018-1279-1 (PMC6102893; doi:10.1186/s12974-018-1279-1)

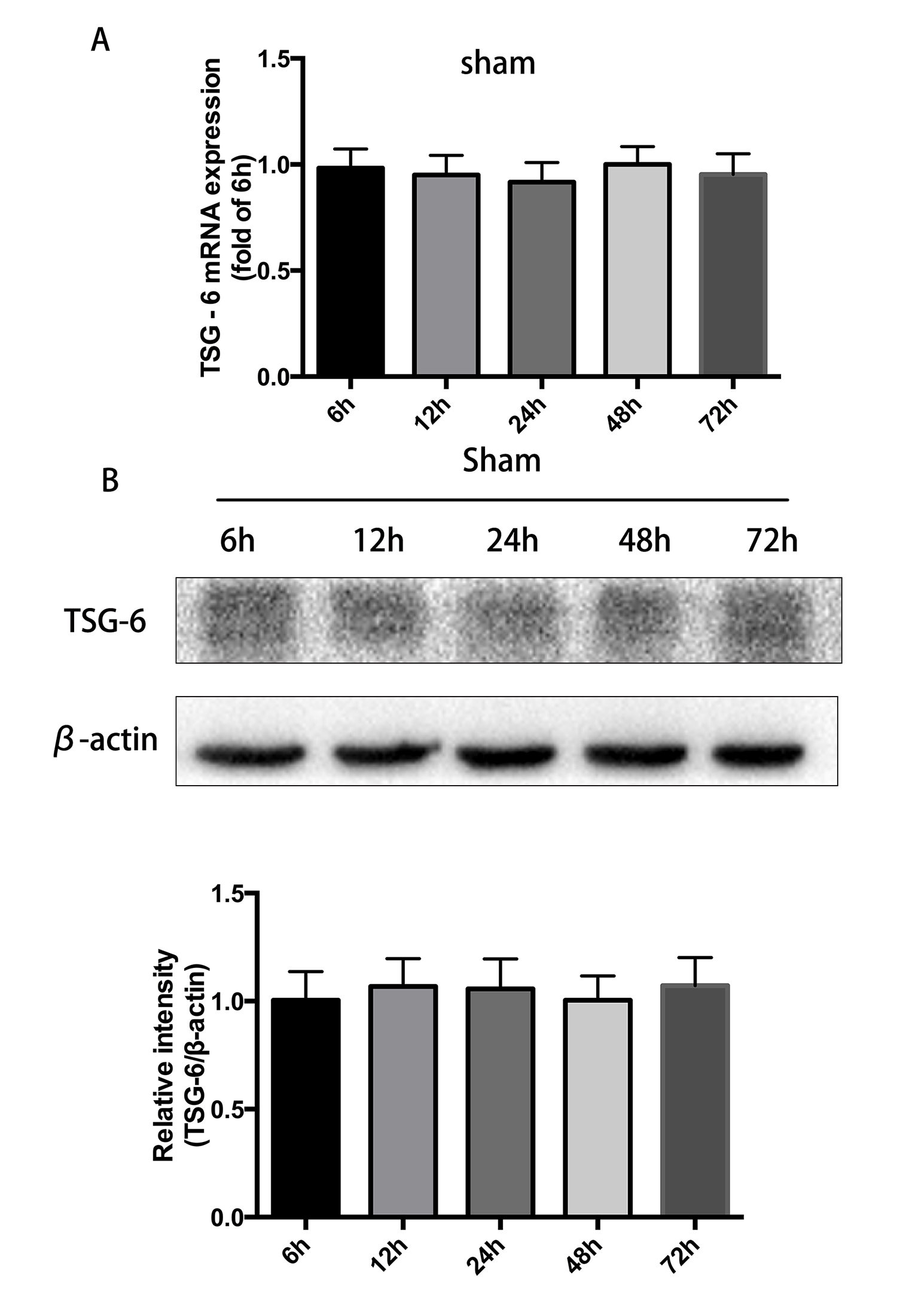

Supplement: Supplementary file 1 — Analysis of levels of TSG-6 gene and protein in different time-points in the sham (6 h, 12 h, 24 h, 48 h, 72 h). No significant differences of TSG-6 gene (A) and protein (B) were found among different groups. All values are presented as means ± SD, n = 4 in each time point per group. (TIF 532 kb) [file 12974_2018_1279_MOESM1_ESM.tif]

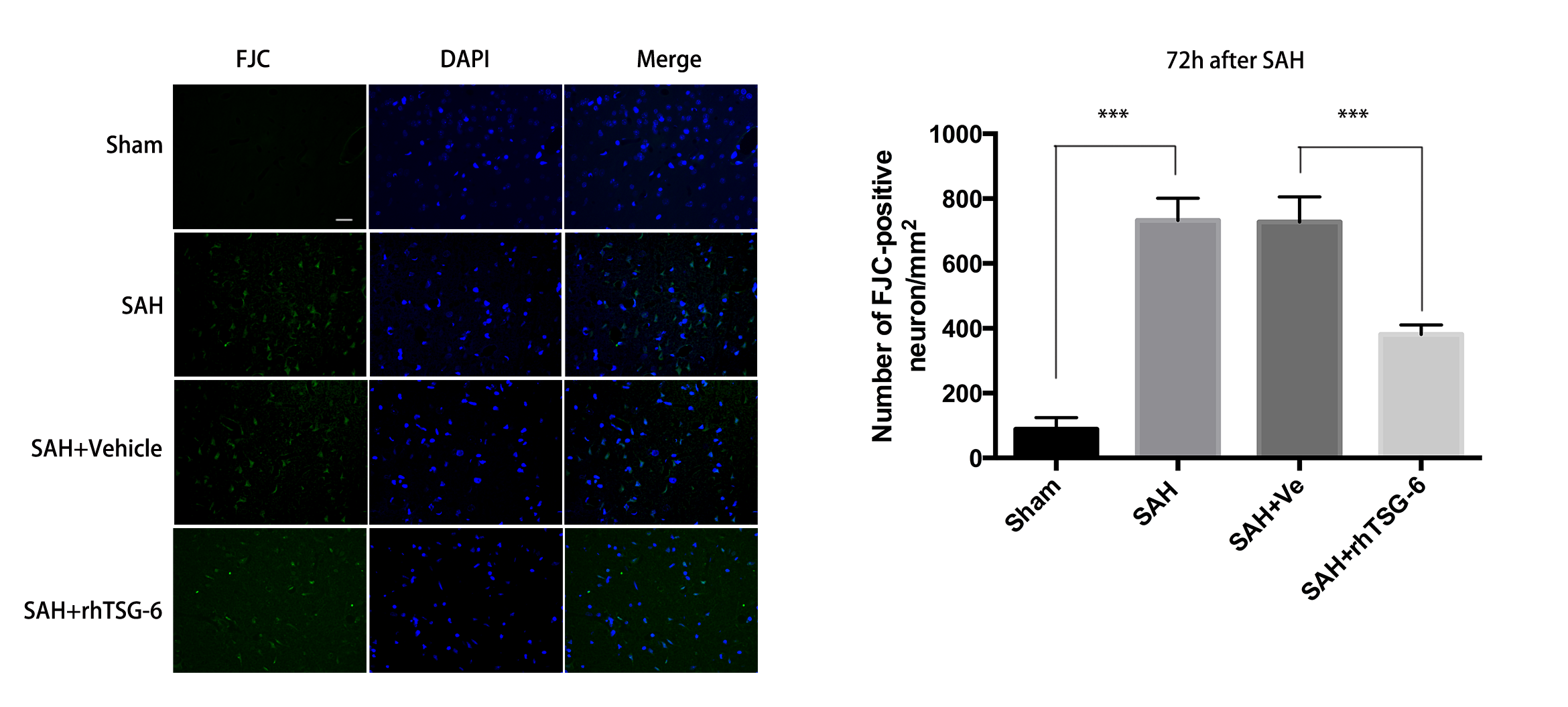

Supplement: Supplementary file 2 — Effects of rh-TSG-6 on neuronal degenerating at 72 h after subarachnoid hemorrhage (SAH). Representative microphotographs and quantitative analysis of Fluoro-Jade C staining (FJC)-positive neurons in the sham, SAH, vehicle and rh-TSG-6 groups at 72 h following operation. n = 6 in each group. Data are expressed as mean ± SD. ***P < 0.001. Scale bar = 20 μm. (TIF 856 kb) [file 12974_2018_1279_MOESM2_ESM.tif]

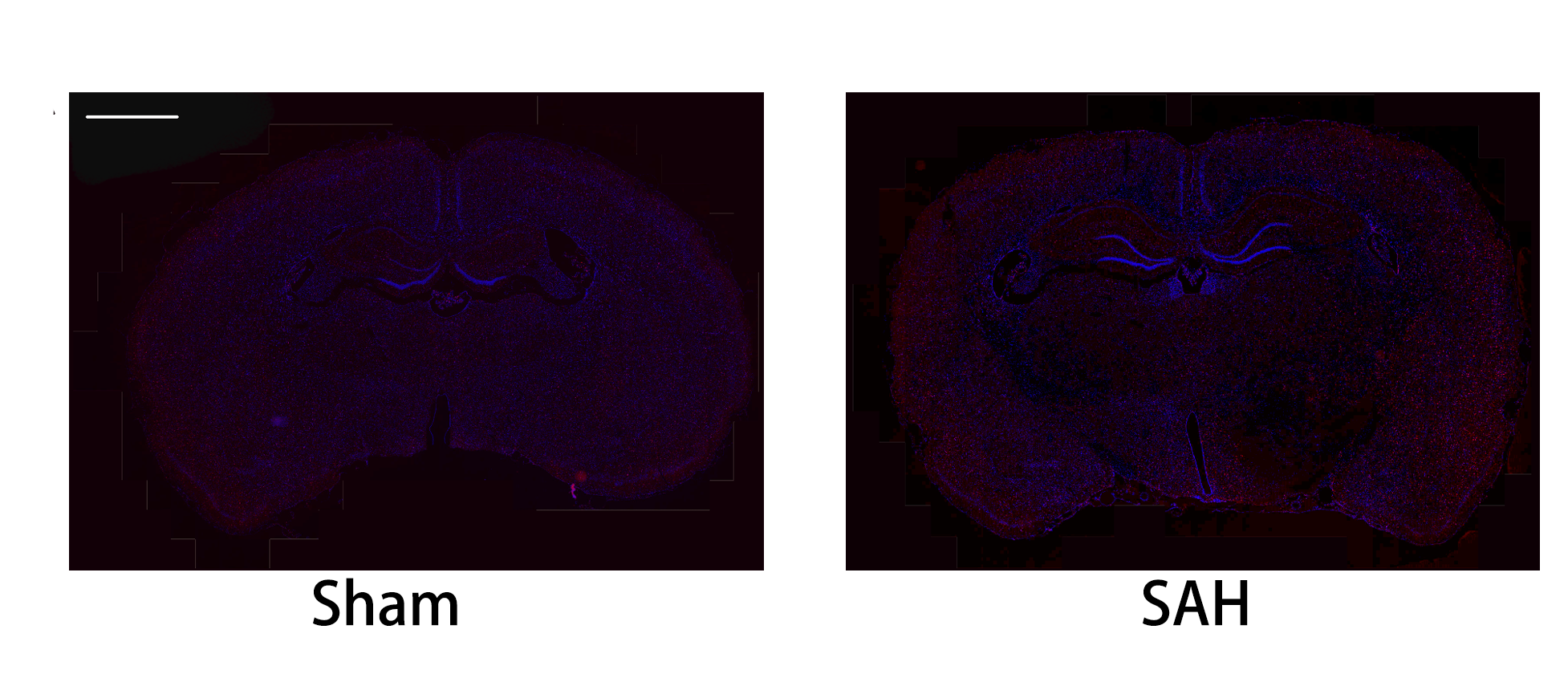

Supplement: Supplementary file 3 — Microglial phenotypic characteristics after SAH induction. Photomicrograph of microglia(red) and DAPI(blue) double immunostaining in a coronal section of the whole brain in sham and SAH groups. n = 5 in each group. Scale bar = 2000 μm. (TIF 1507 kb) [file 12974_2018_1279_MOESM3_ESM.tif]
